# Homeostatic Function and Inflammatory Activation of Ileal CD8^+^ Tissue-Resident T Cells Is Dependent on Mucosal Location

**DOI:** 10.1016/j.jcmgh.2021.06.022

**Published:** 2021-07-02

**Authors:** Lisanne Lutter, Britt Roosenboom, Eelco C. Brand, José J. ter Linde, Bas Oldenburg, Ellen G. van Lochem, Carmen S. Horjus Talabur Horje, Femke van Wijk

**Affiliations:** 1 Centre for Translational Immunology, University Medical Centre Utrecht, Utrecht, the Netherlands; 2 Department of Gastroenterology and Hepatology, University Medical Centre Utrecht, Utrecht, the Netherlands; 3 Department of Gastroenterology and Hepatology, Rijnstate Hospital, Arnhem, the Netherlands; 4 Department of Microbiology and Immunology, Rijnstate Hospital, Arnhem, the Netherlands

**Keywords:** CD8^+^ Tissue-Resident T Cell, Gut Compartmentalization, Transcriptome, Anti-T Cell Trafficking Agents, BSA, bovine serum albumin, CD, Crohn’s disease, CRP, C-reactive protein, FCS, fetal calf serum, FDR, false discovery rate, GzmK, granzyme K, HBI, Harvey-Bradshaw index, HBSS, Hank’s Balanced Salt Solution, HC, healthy control, IBD, inflammatory bowel disease, IL, interleukin, IMC, imaging mass cytometry, log2FC, log2 fold change, NES, normalized enrichment score, PBST, phosphate-buffered saline containing 0.1% Tween-20, TNF, tumor necrosis factor, Trm, tissue-resident memory T cell

## Abstract

**Background & Aims:**

Tissue-resident memory T (Trm) cells, both of the CD4 and CD8 lineage, have been implicated in disease flares in inflammatory bowel disease. However, data are conflicting regarding the profile of human CD8^+^ Trm cells, with studies suggesting both proinflammatory and regulatory functions. It is crucial to understand the functional profile of these cells in the context of (new) therapeutic strategies targeting (trafficking of) gut Trm cells.

**Methods:**

Here, we performed imaging mass cytometry, flow cytometry, and RNA-sequencing to compare lamina propria and intraepithelial CD103^+/–^CD69^+^CD8^+^ Trm cells in healthy control subjects and patients with active ileal Crohn’s disease.

**Results:**

Our data revealed that lamina propria CD103^+^CD69^+^CD8^+^ T cells have a classical Trm cell profile with active pathways for regulating cell survival/death and cytokine signaling, whereas intraepithelial CD103^+^CD69^+^CD8^+^ T cells display tightly regulated innate-like cytotoxic profile. Furthermore, within lamina propria CD8^+^CD103^–^ Trm cells, an Itgb2^+^GzmK^+^KLRG1^+^ population distinct from CD103^+^ CD8^+^ Trm cells is found. During chronic inflammation, especially intraepithelial CD103^+^CD69^+^CD8^+^ T cells displayed an innate proinflammatory profile with concurrent loss of homeostatic functions.

**Conclusions:**

Altogether, these compartmental and inflammation-induced differences indicate that therapeutic strategies could have a different impact on the same immune cells depending on the local compartment and presence of an inflammatory milieu, and should be taken into account when investigating short- and long-term effects of new gut T cell–targeting drugs.

**Summary**

We demonstrate that the human CD8 gut tissue-resident T cell profile is mostly driven by compartmentalization, with IBD inducing a functional shift of these cells primarily in the epithelium. This may have important consequences for T cell trafficking targeting therapies.

Inflammatory bowel disease (IBD), comprising Crohn’s disease (CD) and ulcerative colitis, is a chronic relapsing-remitting inflammatory disease. To date, there is no cure for IBD; therefore, long-term administration of maintenance therapy is often necessary. Recently, a novel class of drugs has been added to the therapeutic armamentarium for IBD, namely compounds that modulate lymphocyte trafficking such as vedolizumab (anti-integrin α4β7) and natalizumab (anti-integrin α4). Another anti-integrin, etrolizumab (anti-integrin β7), is currently in phase III trials.^1^^,^^2^ Expression of integrins enables homing of immune cells to tissues, with integrin α4β7 being the primary gut homing receptor.^3^ Upon localization to the gut, the integrin β7 monomer can dimerize with integrin αE (CD103). Upregulation of CD103 enables T cells to bind to E-cadherin, expressed by epithelial cells, thereby facilitating their intraepithelial retention.^4^, ^5^, ^6^ CD4^+^ T cells are more abundant in the lamina propria, while T cells in the epithelium are primarily of the CD8^+^ lineage.^4^ T cells homed to the lamina propria and epithelium can become tissue-resident memory T (Trm) cells upon expression of the Trm cell markers CD69 and CD103.^7^ Local cues, distinct for the lamina propria and epithelium, might induce further environment-adapted specialization of these Trm cells (CD69^+^CD103^+/–^).^8^, ^9^, ^10^

Recently, it has been suggested that lamina propria CD4^+^ and CD8^+^ CD69^+^CD103^+^ Trm cells might be implicated in disease flares in IBD,^11^ which implies that targeting these cells in IBD could be beneficial. A proinflammatory profile of colonic CD4^+^CD103^+^ T cells in IBD flares has been observed,^12^^,^^13^ but the functional profile of intestinal CD8^+^CD103^+^ T cells is still not completely elucidated.^14^, ^15^, ^16^ Interestingly, in mice adoptive transfer of CD8^+^CD103^+^ T cells reduced the severity of ileitis.^17^ Furthermore, we have previously shown that mucosal CD8^+^CD103^+^ T cell percentages in humans decrease by approximately 40% during CD flares compared with healthy control subjects, and normalize upon achieving remission.^18^^,^^19^ These findings raise the question whether these cells have a proinflammatory or regulatory function. To determine compartmental differences and the functional profile of intestinal CD8^+^ Trm cells, we performed flow cytometry, imaging mass cytometry, and RNA-sequencing on lamina propria and intraepithelial CD103^+^ (and CD103^–^) CD69^+^CD8^+^ T cells in healthy control subjects and patients with active ileal CD.

## Results

**Figure 1:**
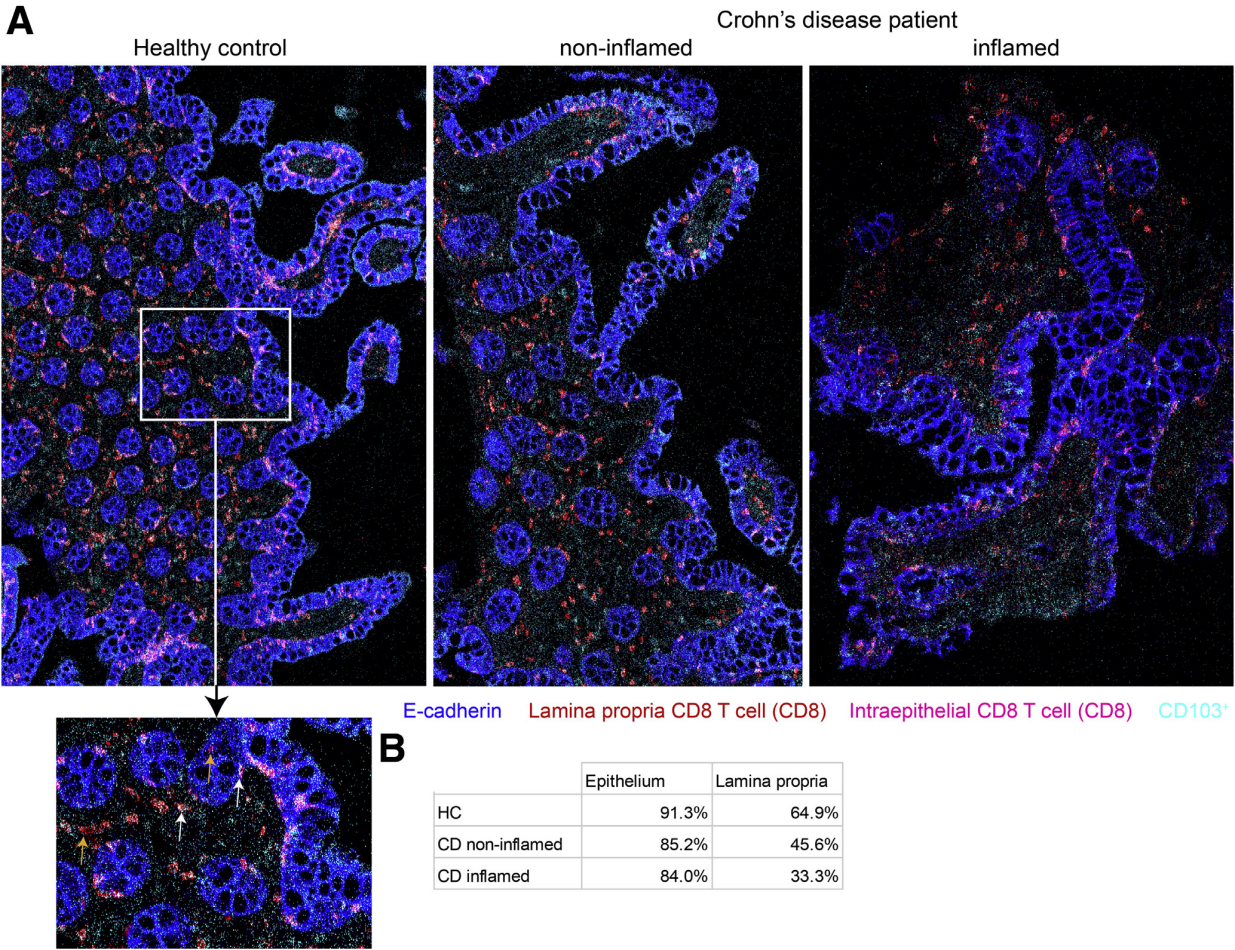
**Visualization of CD103**^**–**^**and CD103**^**+**^**CD8**^**+**^**T cells in the human ileum.** (*A*) Representative composite images of imaging mass cytometry performed on human ileum sections showing an overlay of E-cadherin (blue), CD8 (red in lamina propria, pink in epithelium), and CD103 (cyan, colors white in CD8 T cells) for a healthy control (HC) subject (left), noninflamed ileum of a CD patient (middle), and inflamed ileum of a CD patient (right). The magnified section of the HC subject shows an example of CD103^+^ (white arrows) and CD103^–^ (orange arrows) CD8^+^ T cells within the lamina propria and epithelium. HC subjects: n = 2; CD patients: n = 3 (paired). (*B*) Quantification of CD103^+^ within CD8^+^ T cells in both the epithelium and lamina propria of the HC, CD noninflamed, and CD inflamed ileum. Every value is an average of 2 (HC subjects and noninflamed CD patients) or 3 (inflamed CD patients) samples measured using imaging mass cytometry. For each of the samples, 2 independent counts were performed.

We determined the localization of ileal CD8^+^CD103^+^ T cells in healthy control subjects and patients with *de novo* CD with imaging mass cytometry (IMC). We observed a decrease in percentage of CD103^+^ cells of total CD8^+^ T cells in both the epithelium and lamina propria of CD patients compared with healthy control ileum (Figure 1*A*). This decrease was most pronounced in the lamina propria with, on average, a 50% reduction in CD103^+^ CD8^+^ T cells compared with a 10% decrease in the epithelium (Figure 1*B*). Upon presence of inflammation in CD patients, there was an additional 30% decrease in CD103^+^ CD8 T cells in the lamina propria, whereas in the epithelium CD8^+^ T cells remained predominantly CD103^+^ (Figure 1*B*). Furthermore, there was an absolute decrease in CD8^+^ and consequently CD8^+^CD103^+^ T cells per μm^2^ in both the epithelium and lamina propria of CD patients compared with healthy control subjects (average of 1 CD8^+^ T cell per 1051 μm^2^ in human control subjects, per 2249 μm^2^ in noninflamed CD patients, and per 2589 μm^2^ in inflamed CD patients for the epithelium, and per 839, 1848, and 1957 μm^2^, respectively, for the lamina propria).

**Figure 2:**
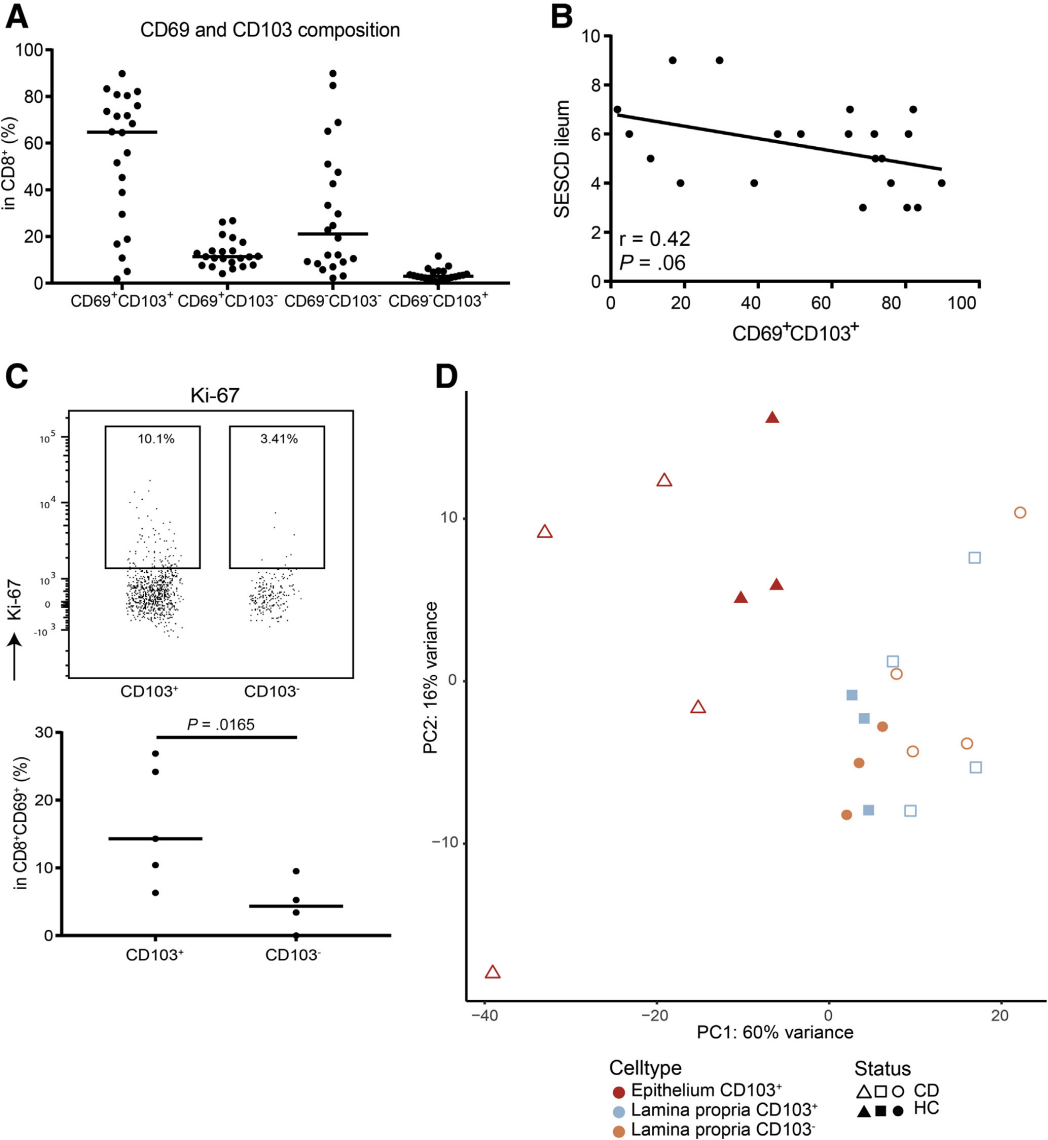
**Characterizing human intestinal CD103**^**–**^**and CD103**^**+**^**CD69**^**+**^**CD8**^**+**^**T cells.** (*A*) Distribution of CD69^+/–^ and CD103^+/–^ CD3^+^CD8^+^ T cells within the ileum of patients with active Crohn CD (n = 21) at time of endoscopy, characterized by flow cytometry. (*B*) Scatterplot and fitted linear regression of the simple endoscopic score for CD (SES-CD) for the ileum and the percentage of total mucosal CD8^+^CD69^+^CD103^+^ T cells derived from inflamed ileum of patients with active CD (n = 21), characterized by flow cytometry. Pearson’s r and the corresponding *P* value are depicted in the graph. (*C*) Representative gating strategy (upper) and quantification (lower) of Ki-67 in both CD103^–^ and CD103^+^ CD69^+^CD8^+^ T cells in patients with active ileal CD (n = 4). The bar represents the median. Comparison was performed with a paired 2-tailed *t* test. (*D*) Unsupervised principal component analysis of all CD69^+^CD8^+^ T cell subsets analyzed by RNA-sequencing; CD103^–^ from the lamina propria (orange/circle) and CD103^+^ from the lamina propria (blue/square) and epithelium (red/triangle) from both healthy control (HC) subjects (closed symbols) and CD patients (open symbols). PC, principal component.

Flow cytometry analysis of ileal CD8^+^ Trm cells in untreated, *de novo* CD (n = 21) (Figure 2*A*) showed a negative trend between the proportion of CD8^+^CD69^+^CD103^+^ T cells and the simple endoscopic score for CD of the ileum (Figure 2*B*). No correlation with other clinical parameters including the Harvey-Bradshaw index, fecal calprotectin, or C-reactive protein was found. In addition, we observed a higher proportion of dividing CD8^+^CD69^+^CD103^+^ compared with CD103^–^ T cells in these untreated, *de novo* CD patients (average of 16.4% and 4.5% Ki-67^+^ cells, respectively) (Figure 2*C*), indicating that CD8^+^CD69^+^CD103^+^ T cells are activated during inflammation.

To investigate the transcriptional profile of gut Trm cells, we performed RNA-sequencing on flow cytometry-based sorted lamina propria and intraepithelial CD103^+^ (and CD103^–^) CD69^+^CD8^+^ T cells in healthy control subjects and patients with active ileal CD. Unsupervised principal component analysis revealed that samples primarily cluster based on the compartment of residence. This relative compartmentalization was less evident during inflammation and for CD103^–^ T cells (Figure 2*D*).

### Lamina Propria CD8^+^CD69^+^CD103^–^ and CD8^+^CD69^+^CD103^+^ T Cells Have Distinct Profiles

**Figure 3:**
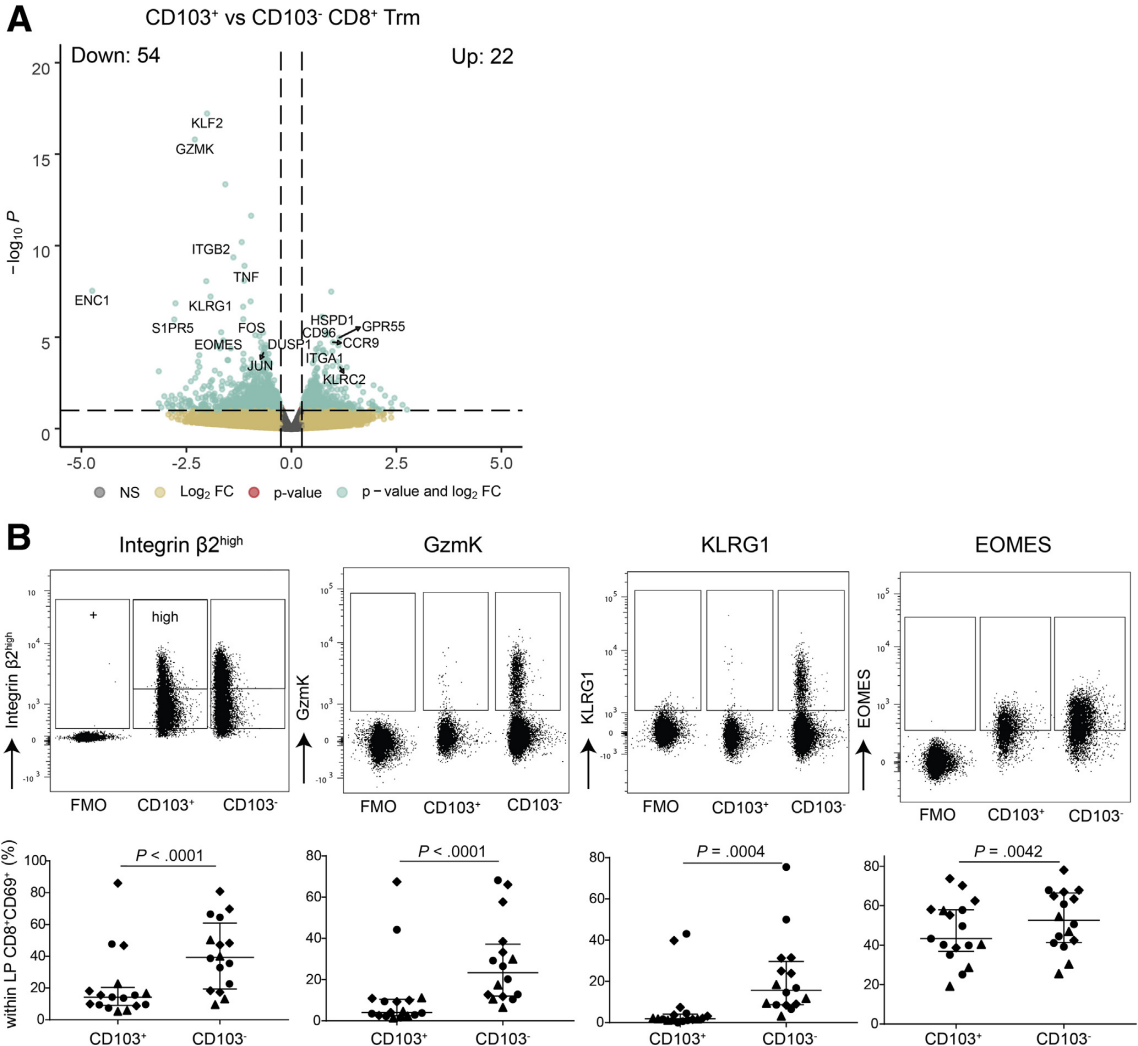
**Subset defining genes of lamina propria CD8**^**+**^**CD69**^**+**^**CD103**^**–**^**and CD8**^**+**^**CD69**^**+**^**CD103**^**+**^**T cells.** (*A*) Volcano plot of the expressed genes, with a nominal *P* value <0.99, comparing lamina propria CD8^+^CD69^+^CD103^+^ to CD8^+^CD69^+^CD103^–^ T cells; selected genes are highlighted. On the x-axis, the log2 fold change (log2FC) is shown, and on the y-axis, the -log10 *P* value (-log10*P*) is shown. Gray indicates not significantly differentially expressed genes, yellow indicates genes with a log2FC >0.25 and -log10*P* > 10 × 10^–2.5^, green indicates genes with a log2FC >0.25 and -log10*P* < 10 × 10^–2.5^. (*B*) Representative flowcytometric dotplots, including Fluorescence Minus One (FMO) control, of Itgb2, GzmK, KLRG1, and EOMES (upper row) and quantification of the respective marker (lower row) comparing lamina propria CD8^+^CD69^+^CD103^+^ and CD103^–^ T cells in healthy control subjects (n = 6–7; circles), CD patients from inflamed (diamonds) and noninflamed (triangles) ileum (paired, n = 4–6). Bars represent median and interquartile range. Comparison was performed with a paired 1-tailed *t* test. NS, not significant.

We first compared the transcriptional profile of CD103^+^ and CD103^–^ CD8^+^ Trm cells (CD69^+^) in the lamina propria. Differential gene expression revealed 22 upregulated and 54 downregulated genes between CD103^+^ and CD103^–^ CD8^+^ Trm cells (false discovery rate [FDR] < 0.1) (Supplementary Table 1). These were shared by inflamed (CD) and noninflamed (healthy control) ileum. *KLF2*, *ENC1*, *GZMK*, *KLRG1*, and *S1PR5* genes known to be downregulated in Trm cells^7^ were also downregulated in CD103^+^CD8^+^ T cells compared with CD103^–^CD8^+^ T cells, whereas *EOMES,* a T effector memory–associated transcription factor,^20^ was upregulated in CD103^–^CD8^+^ T cells (Figure 3*A*). In line with a more differentiated Trm cell phenotype, CD103^+^ CD8^+^ Trm cells also expressed higher levels of *CD160*, *CD96*, and *KLRC2* (encoding NKG2C) (Figure 3*A*). On the protein level, a lower expression of integrin β2 (Itgb2), GzmK, KLRG1, and EOMES on CD8^+^CD103^+^ T cells compared to CD8^+^CD103^–^ T cells was confirmed (Figure 3*B*). These data indicate that CD103^+^ CD8^+^CD69^+^ lamina propria T cells express a less cytotoxic but more pronounced classical Trm cell profile compared with their CD103^–^ counterpart.

**Figure 4:**
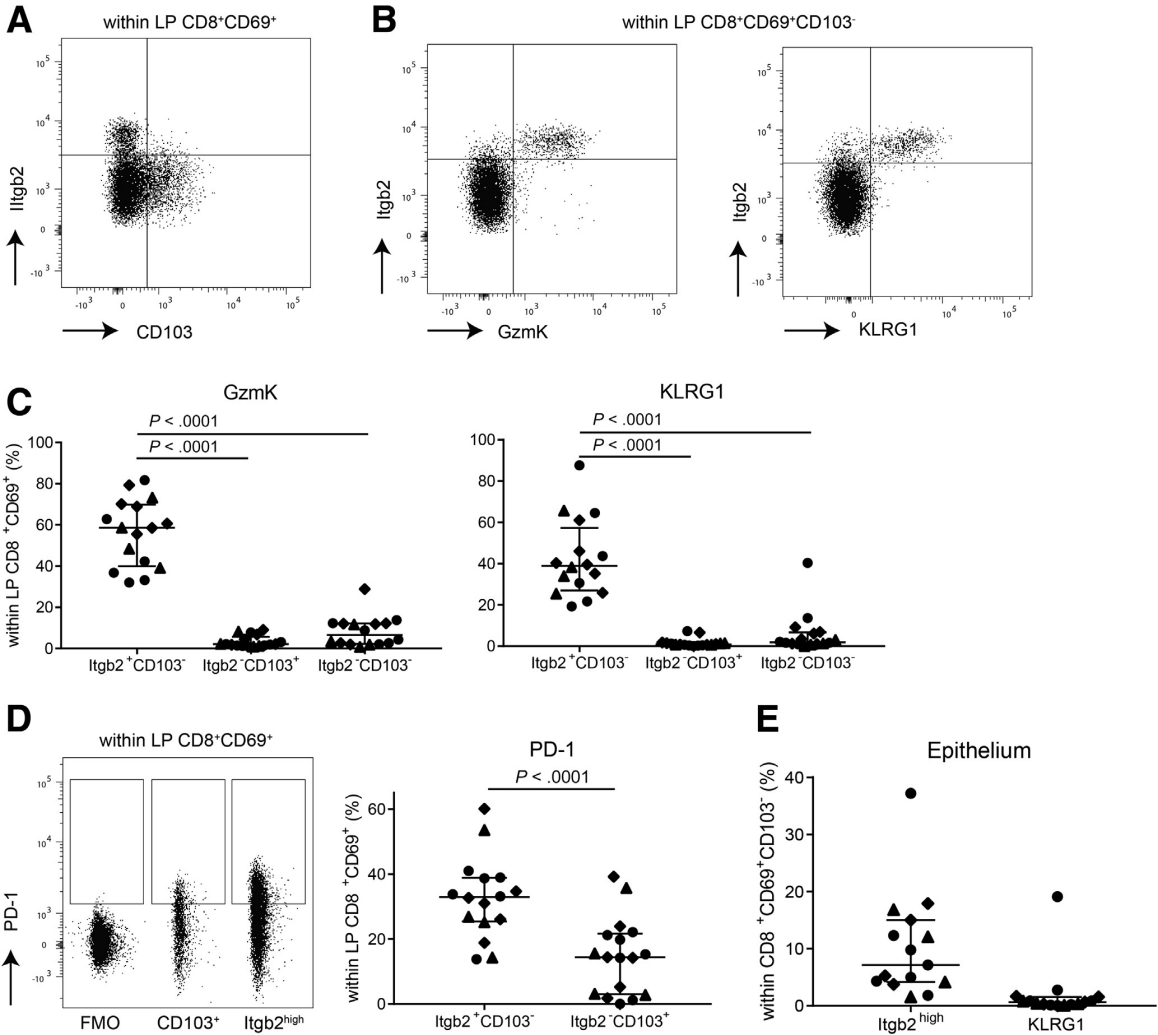
**CD8**^**+**^**CD69**^**+**^**CD103**^**–**^**T cell characterization.** (*A*) Representative flow dotplot of Itgb2^high^ and CD103^+^ expression within lamina propria (LP) CD8^+^CD69^+^ T cells. (*B*) Representative flow dotplot of Itgb2^high^ and GzmK (left) and KLRG1 (right) coexpression within LP CD8^+^CD69^+^CD103^–^ T cells. (*C*) Quantification of GzmK and KLRG1 within Itgb2^+/high^CD103^–^, Itgb2^–/low^CD103^+^, and Itgb2^–/low^CD103^–^ LP CD8^+^CD69^+^ T cells for healthy control subjects (n = 6–7; circles) and CD patients from inflamed (diamonds) and noninflamed (triangles) ileum (paired, n = 4–6). Bars represent median and interquartile range. Comparison was made with a 1-way analysis of variance. (*D*) Representative flow dotplot including Fluorescence Minus One (FMO) control and quantification of PD-1 within Itgb2^high^CD103^–^ and Itgb2^–/low^CD103^+^ LP CD8^+^CD69^+^ T cells. Symbols and n as per panel *C*. Bars represent median and interquartile range. Comparison was made with a paired 2-tailed *t* test. (*E*) Quantification of Itgb2^high^ and KLRG1 within epithelial CD8^+^CD69^+^CD103^–^ T cells. Symbols as per panel *C*. Healthy control subjects: n = 7; CD patients: n = 4. Bars represent median and interquartile range.

Within the CD8^+^CD69^+^CD103^–^ Trm cells compartment, a relatively high heterogeneity was found based on the protein expression data, with <50% Itgb2^high^, GzmK, and KLRG1 expressing cells (Figure 3*B*). Itgb2^high^ expression was almost mutually exclusive with CD103 expression (Figure 4*A*), and expression of GzmK and KLRG1 was predominantly confined to the Itgb2^high^ subset (Figure 4*B* and *C*). Similar to CD103^+^ CD8^+^CD69^+^ Trm cells, CD8^+^ Trm cells lacking CD103 and Itgb2^high^ were mostly GzmK and KLRG1 negative (on average 91.8% and 94.2%, respectively) (Figure 4*C*). Additionally, PD-1 expression, often associated with clonal expansion of CD8^+^ T cells,^21^^,^^22^ was higher in the Itgb2^high^ compared with CD103^+^ CD8^+^ Trm cells (average of 32.7 vs 14.7%) (Figure 4*D*). In summary, within CD103^–^ CD8^+^ Trm cells an Itgb2^high^ Trm cell population characterized by GzmK, KLRG1, and PD-1 is found.

The dichotomy between CD103^+^ and CD103^–^ CD8^+^CD69^+^ Trm cells was not found in the epithelial layer. Itgb2^high^ CD8^+^CD69^+^CD103^–^ Trm cells constituted on average 8.9% of epithelial CD8^+^ T cells, and KLRG1^+^ CD8^+^CD69^+^CD103^–^ Trm cells comprised 1.9% of CD8^+^ T cells in the epithelium (Figure 4*E*).

### The Transcriptional Profile of Ileal CD8^+^CD69^+^CD103^+^ T Cells Is Largely Dependent on Mucosal Localization

**Figure 5:**
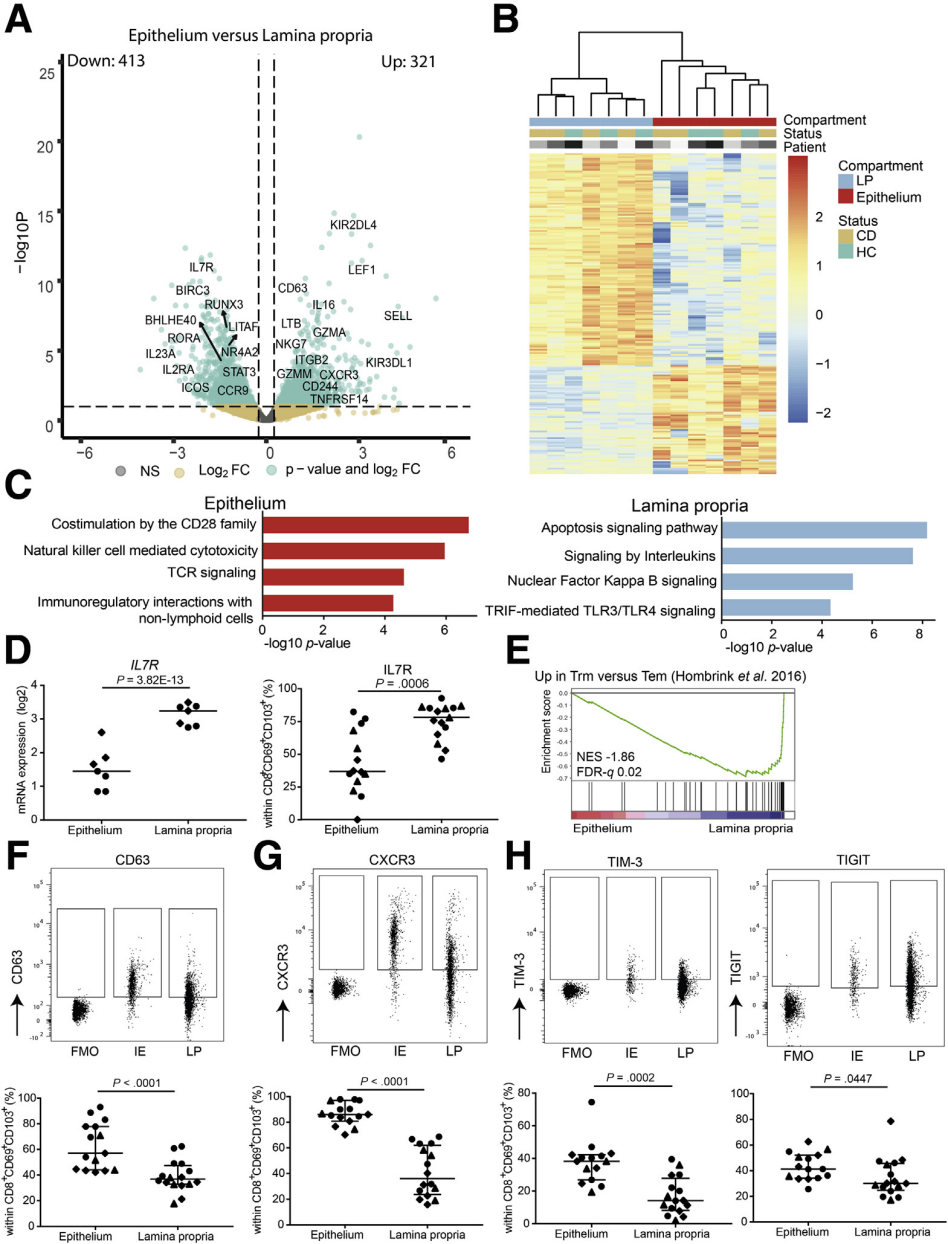
**Location shapes the profile of intestinal CD103**^**+**^**CD69**^**+**^**CD8**^**+**^**T cells.** (*A*) Volcano plot of the expressed genes, with a nominal *P* value <0.99, comparing intraepithelial to lamina propria (LP) CD8^+^CD69^+^CD103^+^ T cells; selected genes are highlighted. On the x-axis, the log2 fold change (log2FC) is shown, and on the y-axis, the -log10 *P* value (-log10*P*) is shown. Gray indicates not significantly differentially expressed genes, yellow indicates genes with a log2FC >0.25 and -log10*P* >10 × 10^–2.5^, green indicates genes with a log2FC > 0.25 and -log10*P* < 10 × 10^–2.5^. (*B*) Heatmap of the top 200 differentially expressed genes comparing CD103^+^ intraepithelial and lamina propria T cells with hierarchical clustering on the columns concerning compartment, status, and patient. Rows are *z* score normalized. (*C*) Pathway terms related to the 321 genes upregulated in intraepithelial (top) and 413 genes upregulated in LP (bottom) CD8^+^CD69^+^CD103^+^ T cells. (*D*) Messenger RNA (mRNA) expression (log2 counts; right) and percentage (left) of ileal CD8^+^CD69^+^CD103^+^ T cells expressing IL7R (CD127) in healthy control subjects (n = 3–5; diamonds) and CD patients from inflamed (circles) and noninflamed (triangles) ileum (paired, n = 4–5). Comparison was performed with Wald’s statistic and a paired 2-tailed *t* test, respectively. (*E*) Gene set enrichment analysis of Trm genes in humans (identified by Hombrink et al)^25^ in pairwise comparisons involving intraepithelial and LP CD8^+^CD69^+^CD103^+^ T cells derived from the ileum of healthy adult control subjects and CD patients pooled, represented by the normalized enrichment score and FDR statistical value (FDRq). (*F*) Representative gating strategy including Fluorescence Minus One (FMO) control (upper panel) and quantification (lower panel) of CD63 in CD8^+^CD69^+^CD103^+^ T cells comparing epithelium (IE) and LP. Bars represent median and interquartile range. Comparison was performed with a paired 1-tailed test. Symbols as per panel *D*. Healthy control subjects: n = 7; CD patients: n = 3–6. (*G*) As per panel *F* but for CXCR3. (*H*) As per panel *F* but for TIM-3 (left) and TIGIT (right). Comparison was performed with a paired 2-tailed *t* test. NS, not significant.

Next, we focused on the differences of CD8^+^CD103^+^ Trm cell profiles, based on their spatial distribution. Differential gene expression of intraepithelial compared with lamina propria CD8^+^CD69^+^CD103^+^ T cells revealed 321 upregulated and 413 downregulated genes shared by CD patients and healthy control subjects (FDR < 0.1) (Figure 5*A* and *B*; Supplementary Table 1). Subsequent pathway analysis showed enrichment of T cell receptor signaling, cytotoxicity, and interaction with nonimmune cells in the epithelial compartment (Figure 5*B*, left panel). In the lamina propria, cytokine signaling and inhibition of apoptosis were enriched (Figure 5*B*, right panel). In support of the latter, expression of the interleukin (IL)-7 receptor, which is essential for T cell homeostasis and long-term survival,^23^^,^^24^ was upregulated at both messenger RNA and protein level in lamina propria CD8^+^CD69^+^CD103^+^ T cells (Figure 5*D*). Furthermore, classical CD8^+^ Trm cell genes, including *RUNX3*, *NR4A2*, *ICOS*, and *LITAF*, showed higher expression in lamina propria CD8^+^CD69^+^CD103^+^ T cells (Figure 5*A*). The more profound Trm cell profile of lamina propria compared with intraepithelial CD8^+^CD69^+^CD103^+^ T cells was also supported by gene set enrichment analyses for a core Trm cell gene set (Figure 5*E*).^25^ However, intraepithelial CD8^+^CD69^+^CD103^+^ T cells did not show enrichment of core effector memory or central memory T cell–related gene sets. We did observe elevated expression of cytotoxic genes such as *NKG7*, *GZMM*, *LTB, GZMA*, and killer-immunoglobulin receptors (*KIR2DL4*, *KIR3DL1*, *KIR2DS4*) (Figure 5*A*) in the epithelial subset. Even though KIRs were elevated on messenger RNA level, no difference for the inhibitory KIRs was observed on protein level, and intraepithelial CD8^+^ T cell KIR expression was low overall (average KIR3DL1 expression of 3.8% in the epithelium). Elevated expression of CD63 was observed in intraepithelial compared with lamina propria CD8^+^CD69^+^CD103^+^ T cells on protein level (Figure 5*F*), indicative of secretory vesicles containing cytotoxic proteins. Furthermore, CXCR3 was highly expressed on epithelial CD8^+^CD69^+^CD103^+^ T cells (Figure 5*G*) supporting immunoregulatory interactions with nonlymphoid cells (Figure 5*C*, left panel), as its ligands are expressed by epithelial cells.^26^ In addition, the immune checkpoints TIM-3 (HAVCR2) and TIGIT were more highly expressed by epithelial CD8^+^CD69^+^CD103^+^ T cells (Figure 5*H*). In summary, CD8^+^CD69^+^CD103^+^ T cells in the lamina propria show a more classical Trm cell profile and cytokine signaling, whereas in the epithelium a tightly regulated innate-like cytotoxic profile is more pronounced.

### Different Profiles in Healthy Control Subjects and CD Patients

**Figure 6:**
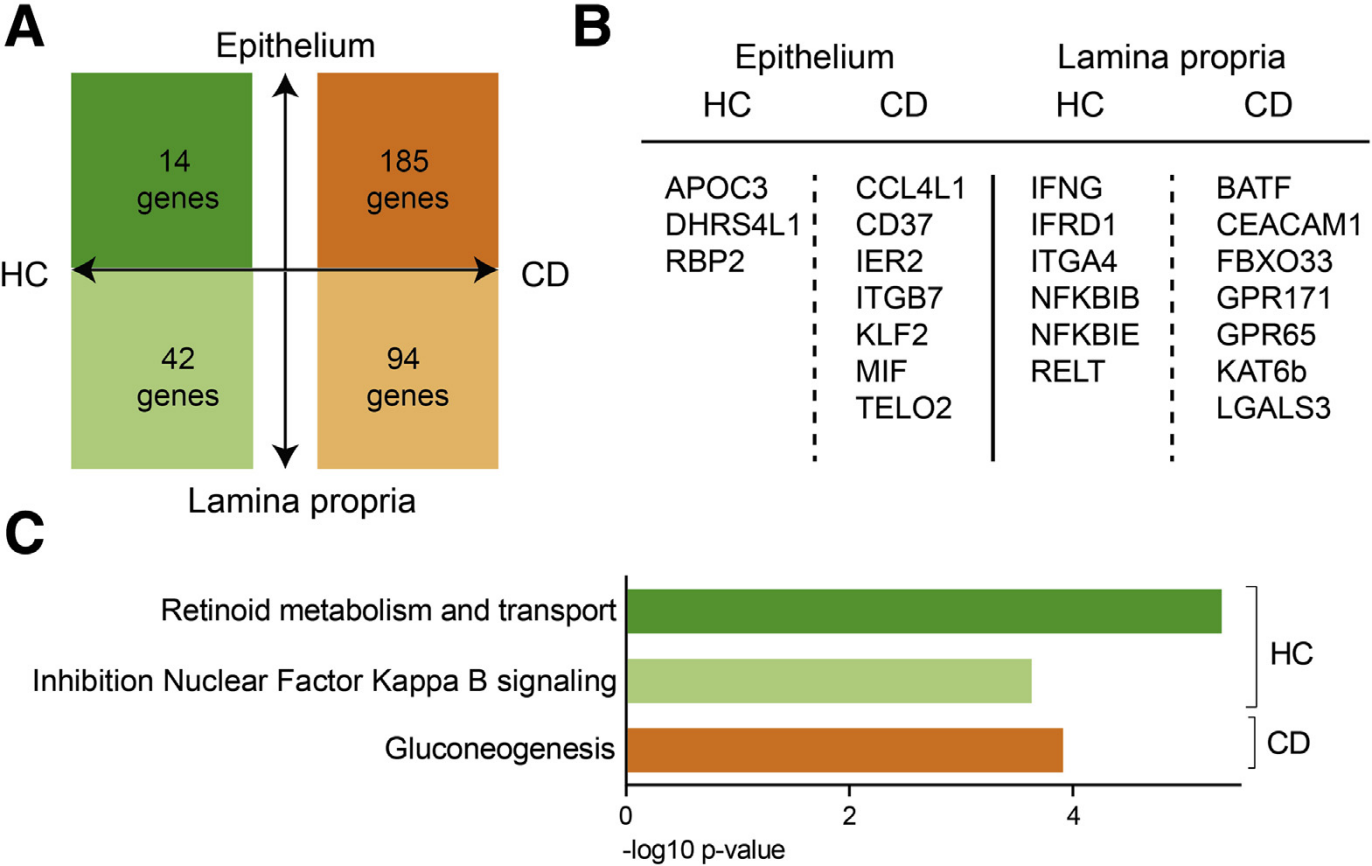
**During inflammation, most upregulated genes are found in CD8**^**+**^**Trm cells from the epithelium.** (*A*) Diagram showing the differentially expressed genes that are specific (ie, not differentially expressed in the other disease state) for healthy control (HC) subjects (left) or CD patients (right). The upper part depicts the number of genes that are upregulated and the lower depicts part those that are downregulated in intraepithelial compared with lamina propria CD8^+^CD69^+^CD103^+^ T cells from the ileum. (*B*) Selection of top genes up- and downregulated as per panel *A*. (*C*) Pathway terms related to the genes specific for CD8^+^ T cells from HC subjects or CD patients for both lamina propria and epithelium derived from panel *A* (colors reflect the colors of panel *A*).

Besides compartmental differences, an inflammatory milieu can influence the transcriptomic and functional profile of tissue T cells. Transcriptional differences between CD103^+^ and CD103^–^ CD8^+^ Trm cells in the lamina propria induced by inflammation were minimal. Only *OASL* and *CCL4* were more highly expressed in CD103^–^ compared with CD103^+^ CD8^+^ Trm cells in inflamed ileum of CD patients. Differences between transcriptional profiles of CD8^+^CD69^+^CD103^+^ T cells between healthy control subjects and active CD patients were mainly found in the epithelium (disease-specific genes: 185 genes in the CD epithelium, 94 genes in the CD lamina propria, and 14 and 42 genes for healthy control subjects, respectively) (Figure 6*A*; Supplementary Table 1). CD-specific genes included the innate proinflammatory *IER2* and *MIF* for intraepithelial CD8^+^CD69^+^CD103^+^ T cells, and in the lamina propria, *BATF* and *LGALS3* (encoding Galectin-3), both previously associated as drivers of IBD inflammation (Figure 6*B*).^27^^,^^28^ On the pathway level, there was enrichment of gluconeogenesis in CD patients, whereas in healthy control subjects retinoid (vitamin A) metabolism was enriched in intraepithelial CD8^+^CD69^+^CD103^+^ T cells (Figure 4*C*). Together this indicates that inflammation in CD patients primarily affects the profile of CD8^+^CD69^+^CD103^+^ T cells in the epithelium.

## Discussion

In the present study, we demonstrate that intestinal CD8^+^ Trm cell transcription profiles depend on their mucosal localization. Lamina propria located CD8^+^CD103^+^ T cells have a classical Trm cell profile with active pathways for regulating longevity and cytokine signaling, while intraepithelial CD8^+^CD103^+^ T cells actively sense the external environment as part of the mucosal barrier and display enrichment in natural killer receptors and innate-like markers in line with previous studies in both mice and humans.^29^^,^^30^ The changes seen during active inflammation are more pronounced in the intraepithelial CD8^+^CD103^+^ T cell subset, leading to an innate proinflammatory profile with a concurrent loss of homeostatic functions such as vitamin metabolism. These data support recent observations in ulcerative colitis in which disease-susceptibility loci were mostly enriched in intraepithelial CD8^+^ T cells, especially during active inflammation.^8^ Furthermore, the differences observed between epithelial and lamina propria CD8^+^CD103^+^ Trm cells suggest that potential infiltrating cells from the lamina propria in the epithelial layer also acquire a proinflammatory innate profile. The microenvironment thus has an important role in skewing a cell’s phenotype.

We also describe transcriptomic and protein differences between CD103^–^ and CD103^+^ CD8^+^ Trm cells in the lamina propria, which corroborate and expand upon previous findings in the human intestine.^9^^,^^15^^,^^16^ The CD8^+^CD103^–^ Trm cell subset in the lamina propria was defined by high Itgb2 expression further characterized by PD-1, GzmK, KLRG1, and EOMES. A recent study of donor-derived Trm cells after intestinal transplantation also described 2 transcriptionally different CD8 Trm cell subsets, where the CD8^+^CD69^+^CD103^–^ subset characterized by coexpression of *ITGB2* displayed a more cytotoxic profile compared with the CD8^+^CD69^+^CD103^+^ subset.^16^ Similar findings were recently reported in a study of ileum samples obtained after ileocecal resection in CD patients demonstrating a statistically significant increase in percentages of CD8^+^CD103^–^KLRG1^+^ Trm cells in inflamed compared with noninflamed ileum and healthy control subjects, with no difference for the CD8^+^CD103^+^ subset.^15^ The CD8^+^CD103^–^KLRG1^+^ expressed higher levels of GZMB, whereas CD8^+^CD103^+^ Trm cells expressed higher levels of IL-22, IL-26, and CCL20.^15^

Our data show a decrease in CD8^+^ T cells per μm^2^ in both the epithelium and lamina propria in inflamed ileum of CD patients compared with paired noninflamed ileum and the ileum of healthy control subjects. Additionally, within CD8^+^CD69^+^ T cells, a decrease in CD103^+^ Trm cells and a relative increase of CD103^–^Itgb2^high^KLRG1^+^GzmK^+^ Trm cells was observed in inflamed ileum of CD patients. Recently, Tkachev et al^31^ observed that pathogenic cells in graft-vs-host disease in a simian transplantation model comprise rapidly developed CD8^+^CD69^+^ Trm cells, which were CD103^–^ but expressed *ITGB2*, *CCL4L1*, *CD74*, and *CCL3* among others. Another study recently linked appearance and accumulation of a GZMK^+^ CD8^+^ T cell population to an inflammatory phenotype in immune aging. This subset is characterized by both high PD-1 and TIGIT expression, is clonally expanded, and is regulated by EOMES and BATF.^22^ These data suggest that within the CD103^–^ CD8^+^ Trm cell population in the lamina propria, a CD69^+^CD103^–^Itgb2^high^GzmK^+^KLRG1^+^ Trm cell subset with pathogenic potential is present.

In line with our study, single cell RNA-sequencing of colonic T cells showed presence of multiple CD8^+^ Trm cell clusters, of which a KLRG1^+^EOMES^+^ITGB2^+^ subset is enriched in ulcerative colitis, and the CD103^+^ population in healthy control subjects.^32^ TCR analysis showed overlap between all CD8^+^ Trm cell clusters except for between these distinct CD8^+^ Trm cell subsets.^32^ This was similar to findings for KLRG1^+^CD103^–^ and KLRG1^–^CD103^+^ CD8^+^ T cells in ileal transplant material.^9^ These distinct CD8^+^ Trm cell subsets thus seem to originate from different CD8^+^CD69^+^ T cells. CD103^+^ CD8^+^ Trm cells residing in the epithelium and the lamina propria, however, had similar TCR repertoires,^9^ indicating that they are derived from the same pool.

Lamina propria and epithelial differences, described in the present study, could be partially due to adhesion of CD103 (integrin αE) to E-cadherin, which initiates intracellular signaling to advance effector functions.^33^ The receptor E-cadherin is only expressed in the epithelium, so CD103 expression in the lamina propria could be redundant and therefore exert less influence on the function of the cell. Further fine-tuning of the functional profile is most likely induced by local cues.^4^^,^^7^ Whether the severity of inflammation correlates with the magnitude of CD103^+^CD8^+^ T cell changes, both in number and in functional profile, is unknown.

Etrolizumab (anti-integrin β7) has been shown to be promising in phase II and III clinical trials in IBD.^34^^,^^35^ In vitro, etrolizumab induces internalization of integrin β7, impairing its adhesion to MAdCAM-1, and blocking migration of immune cells to the gut, and has therefore a similar mode of action as the anti-integrin α4β7 antibody vedolizumab.^36^ Additionally, etrolizumab affects the adhesion of integrin αE to E-cadherin resulting in decreasing intraepithelial CD103^+^ cell counts (without distinction for immune cell type)^34^ and in a reduced accumulation of mainly CD8^+^ and T helper 9 cells.^37^ Thus, primarily the CD8^+^CD103^+^ Trm cell subset seems to be targeted by etrolizumab. The question is whether this is desirable because CD4^+^CD69^+^CD103^+^ and not CD8^+^CD69^+^CD103^+^ T cells have been correlated with disease flares in IBD.^11^

The fact that higher *ITGAE* (integrin αE/CD103) counts at baseline as reported in the phase II etrolizumab trial were related to higher therapeutic response rates does not underline a pathogenic role of the CD8^+^CD103^+^ subset, as this was demonstrated in bulk data^34^ and thus reflected a combination of dendritic cells, CD4^+^ cells, and/or CD8^+^ T cells. Additionally, post hoc analysis of the latter study showed that patients with high *ITGAE* counts before treatment had milder disease activity with a lower endoscopic disease score at baseline.^13^ This corresponds with our observation that patients with milder disease, defined as a lower simple endoscopic score for CD of the ileum, had higher mucosal levels of CD8^+^CD103^+^ T cells.

Our findings demonstrate the heterogeneity and dual functionality of Trm cell subsets in the intestinal mucosa. Long-term integrin β7 blockade could have a negative impact on the presence, and thus homeostatic functions, of these CD8^+^CD103^+^ T cells, which clearly warrants further evaluation. For example, CD8^+^ CD103^+^ T cells might contribute to vitamin A metabolism, which is essential in maintaining epithelial integrity.^38^ The suggestion that CD8^+^CD103^+^ T cells in IBD patients in remission regain a regulatory profile^14^ should be further studied in a longitudinal cohort. Altogether, these differences indicate that therapeutic strategies could have a different impact on the same immune cells depending on the compartment of residence and presence of an inflammatory milieu, and should be taken into account when investigating short- and long-term effects of new gut T cell–targeting drugs. In conclusion, the transcriptional profile of CD8^+^ Trm cells differs depending on the degree of inflammation and location within the gut.

## Materials And Methods

### Patient Inclusion

Patients with CD, most newly diagnosed, were prospectively enrolled at the outpatient clinic of the Rijnstate Crohn and Colitis Centre (Arnhem, the Netherlands). During ileocolonoscopy, multiple biopsy specimens were taken for histopathological analysis, for immunophenotyping by flow cytometry analysis (n = 27), and for RNA-sequencing of sorted subsets and imaging mass cytometry (n = 4). Healthy control subjects (n = 10) underwent ileocolonoscopy for polyp surveillance or iron deficiency. They had normal macroscopical ileal mucosa, which was confirmed by histology (see Table 1 for patient characteristics).

**Table 1:** Baseline Patient Characteristics

	Flow Cytometric Analysis		RNA-seq		CyTOF	
	CD Patients (n = 27)	HC Subjects (n = 7)	CD Patients (n = 4)	HC Subjects (n = 3)	CD Patients (n = 3)	HC Subjects (n = 2)
Sex						
Female	16 (76.2)	4 (57.1)	3 (75)	2 (66.7)	2 (66.7)	1 (50)
Male	5 (23.8)	3 (42.8)	1 (25)	1 (33.3)	1 (33.3)	1 (50)
Age, y	24 (20-32)	50 (46–60)	49 (30–54)	36	46	36
Smoking status						
Yes	11 (52.4)	0 (0)	2 (50)	2 (66.7)	2 (66.7)	2 (100)
No	7 (33.3)	7 (100)	0 (0)	1 (33.3)	0 (0)	0 (0)
Ceased	3 (14.3)	0 (0)	2 (50)	0 (0)	1 (33.3)	0 (0)
Duration of complaints before ileocolonoscopy, wk	14 (9–23)	NA	4 (1–6)	NA	4	NA
Calprotectin, μg/g	231 (156–487)	NA	120 (51–728)	NA	139	NA
CRP, mg/L	25 (11–62)	NA	9 (4–19)	NA	4	NA
Treatment at ileocolonoscopy						
None	22 (81.4)	7 (100)	4 (100)	3 (100)	3 (100)	2 (100)
Mesalamine	0 (0)	0 (0)	0 (0)	0 (0)	0 (0)	0 (0)
Steroids	0 (0)	0 (0)	0 (0)	0 (0)	0 (0)	0 (0)
Thiopurine	1 (4.5)	0 (0)	0 (0)	0 (0)	0 (0)	0 (0)
Mesalamine + thiopurine	0 (0)	0 (0)	0 (0)	0 (0)	0 (0)	0 (0)
Anti-TNF	3 (13.6)	0 (0)	0 (0)	0 (0)	0 (0)	0 (0)
Anti-IL12/23	1 (4.5)	0 (0)	0 (0)	0 (0)	0 (0)	0 (0)
HBI score						
<5, remission	2 (7.4)	NA	1 (25)	NA	0 (0)	NA
5–7 mild disease	12 (44.4)		2 (50)		2 (66.7)	
8–16 moderate disease	9 (33.3)		1 (25)		1 (33.3)	
>16 severe disease	4 (14.8)		0 (0)		0 (0)	
SES-CD score						
0–2 inactive disease	0 (0)	NA	0 (0)	NA	0 (0)	NA
3–6 mild disease	8 (29.6)		1 (25)		1 (33.3)	
7–15 moderate disease	12 (44.4)		2 (50)		1 (33.3)	
≥16 severe disease	7 (25.9)		1 (25)		3 (33.3)	
Montreal CD						
Location	10 (37)		3 (75)		2 (66.7)	
L1: ileal	0 (0)	NA	0 (0)	NA	0 (0)	NA
L2: colonic	17 (73)		1 (25)		1 (33.3)	
L3: ileocolonic						
Behavior	23 (85.2)		1 (25)		1 (33.3)	
B1: nonstricturing, nonpenetrating						
B2: stricturing	3 (11.1)		3 (75)		2 (66.7)	
B3: penetrating	1 (3.7)		0 (0)			

Values expressed in n (%) or as median with interquartile range.

CD, Crohn’s disease; CRP, C-reactive protein; HBI, Harvey-Bradshaw index; HC, healthy control; RNA-seq, RNA-sequencing; SES-CD, simple endoscopic score for CD; TNF, tumor necrosis factor.

### Mechanical Cell Isolation

Biopsies for analysis without separation of the lamina propria and epithelium were stored in a phosphate-buffered saline solution at 2–8°C, after which flow cytometric analysis was performed within 8 hours. We carried out mechanical preparation of single cell suspensions. Hereto, specimens were pooled and blended in Hank’s Balanced Salt Solution (HBSS) (Gibco, Waltham, MA) supplemented with 1% bovine serum albumin (BSA) using a 70-μm gaze and spatula followed by Ficoll density gradient centrifugation. The homogenate was resuspended, after washing, in 0.5 mL HBSS/1% BSA.

### Enzymatic Cell Isolation

Biopsies were collected in HBSS media containing 2% fetal calf serum (FCS) and 0.2% amphotericin B. The intestinal tissue was transferred to HBSS supplemented with 1 mM DTT (Sigma-Aldrich, St Louis, MO) and placed on a rolling device for 10 minutes at 4°C. After discarding the supernatant, the intestinal tissue was transferred to HBSS supplemented with 2% FCS and 5 mM EDTA and shaken (2×) at 180 rpm for 30 minutes at 37°C. The tissue suspension was passed through a 70-μm cell strainer (Costar, Greiner Bio-One, Germany) and constituted the intraepithelial population. To obtain lamina propria T cells, intestinal biopsies were subsequently incubated for 1 hour at 37°C with 1 mg/mL Collagenase IV (Sigma-Aldrich) in RPMI medium (supplemented with 10% FCS, 100 U/mL penicillin-streptomycin, and 0.2% amphotericin B), then forcefully resuspended through a 19G needle, washed, and filtered with 70-μm cell strainer (Costar). The cell suspensions were used for RNA-sequencing after sorting different T cell subsets.

### Imaging Mass Cytometry

Intestinal biopsies were fixed in 10% neutral buffered formalin, paraffin-embedded, and 2 slides containing consecutive 4-μm-thick sections of all samples were prepared. One slide was stained with hematoxylin and eosin for histological assessment and the second slide was stained for IMC. IMC combines immunohistochemistry with high-resolution laser ablation of stained tissue sections followed by CyTOF mass cytometry enabling imaging of multiple proteins at subcellular resolution.^39^ All antibodies were conjugated to lanthanide metals (Fluidigm, San Francisco, CA) using the MaxPar antibody labeling kit and protocol (Fluidigm), and eluted in antibody stabilization buffer (Candor Bioscience, Wangen, Germany) for storage.

The slide was baked for 1.5 hours at 60°C, deparaffinized with fresh xylene for 20 minutes, and subsequently rehydrated in descending grades of ethanol (100% [10 minutes], 95%, 80%, and 70% (5 minutes each). After washing for 5 minutes in Milli-Q and 10 minutes in phosphate-buffered saline containing 0.1% Tween-20 (PBST), heat-induced epitope retrieval was conducted in Tris/EDTA (10 mM/1 mM, pH 9.5) for 30 minutes in a 95°C water bath. The slide was allowed to cool to 70°C before washing in PBST for 10 minutes. To decrease nonspecific antibody binding, tissue sections were blocked with 3% BSA and Human TruStain FcX (1:100; BioLegend, San Diego, CA) in PBST for 1 hour at room temperature. The antibody cocktail was prepared by mixing all antibodies at concentrations specific for the assay in PBST+0.5% BSA. After careful removal of the blocking buffer, the slide was incubated overnight at 4°C with the antibody cocktail. Antibodies used were E-cadherin 142Nd (metal tag) (clone 24E10, CST3195BF; Cell Signaling Technology, Danvers, MA), CD103 153Eu (clone EPR4166(2), ab221210; Abcam, Cambridge, United Kingdom), CD8α 162Dy (clone C8/144B, 14-0085-82; Thermo Fisher Scientific, Waltham, MA). Following three 5-minute washes in PBST and rinsing in Milli-Q, the tissue was counterstained with 0.1% toluidine blue for 5 minutes to enable tissue structure visualization under bright field microscopy if desired. Upon washing for 5 minutes in Milli-Q, the slide was incubated with Ir-intercalator (1:500 in PBST; Fluidigm) for 60 minutes at room temperature. Finally, the slide was washed in Milli-Q and air dried for at least for 20 minutes at room temperature.

Images were acquired at a resolution of 1 μm using a Hyperion Imaging System (Fluidigm). Regions of interest were selected based on the hematoxylin and eosin slides after which areas with an approximate size of 1000 × 1000 μm were ablated and acquired at 200 Hz. Pseudo-colored intensity maps were generated of each mass channel. Composite images were created and analyzed for each sample using ImageJ (version 1.47; National Institutes of Health, Bethesda, MD), and any changes to the brightness or contrast of a given marker were consistent across all samples.

### Flow Cytometry

**Figure 7:**
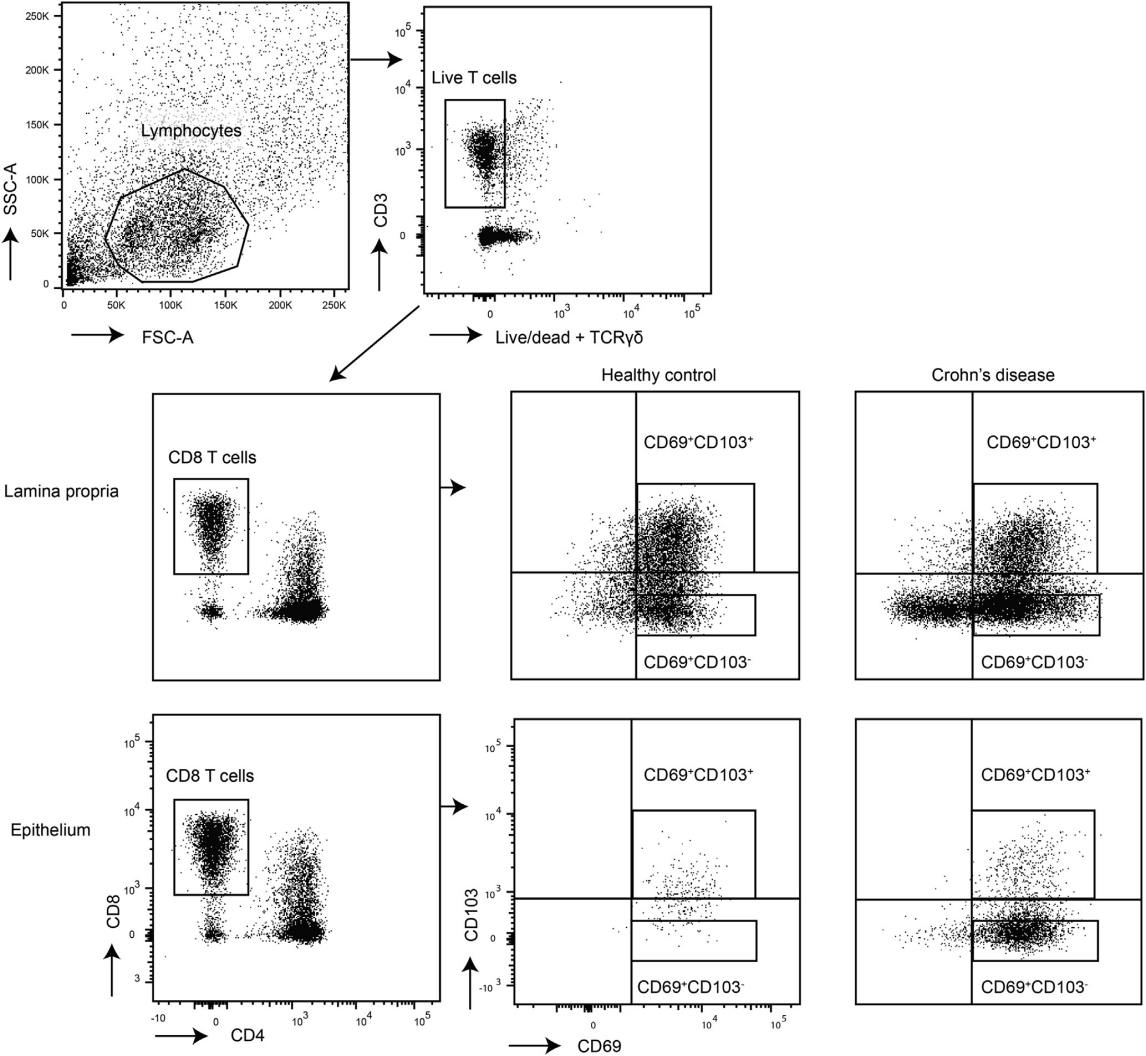
**Gating strategy of intestinal CD103**^**+**^**and CD103**^**–**^**CD69**^**+**^**CD8**^**+**^**T cells.** Gating for fluorescence-activated cell sorting for RNA-sequencing (strict gates) and flow cytometry (quadrant gates).

For flow cytometric analysis, the intestinal cells were incubated with surface antibodies for 20 minutes at 4°C. Antibodies used were fixable viability dye eF506 (65-2860-40; eBioscience, San Diego, CA), anti-human CD3 APC-H7 (clone SK7, 560176; BD Biosciences, Franklin Lakes, NJ), CD8α PerCP-Cy5.5 (clone SK1, 565310; BD Biosciences), CD8α BV650 (clone UCHT1, 563822; BD Biosciences), CD69 PE (clone L78, 555531; BD Biosciences), CD69 PE-Cy7 (clone FN50, 557745; BD Biosciences), CD103 PE (clone Ber-ACT8, 550260; BD Biosciences), CD103 FITC (clone Ber-ACT8, 550259; BD Biosciences), TIM-3 BV711 (clone 7D3, 565566; BD Biosciences), PD-1 BV711 (clone EH12.1, 564017; BD Biosciences), Itgb2/CD18 FITC (clone TS1/18, 302105; BioLegend), CD3 BV605 (clone UCHT1, 300460; BioLegend), KLRG1 PE-CF594 (clone 14C2A07, 368608; BioLegend), CXCR3 BV605 (clone G025H7, 353728; BioLegend), CD3 AF700 (clone UCHT1, 300424; BioLegend), CD63 FITC (clone H5C6, 353006; BioLegend), CD4 BV785 (clone OKT4, 317442; BioLegend), and TCRγδ BV510 (clone B1, 331220; BioLegend), and TIGIT PerCP-eF710 (clone MBSA43, 46-9500-42; eBioscience). For intracellular staining cells were fixed and permeabilized using eBioscience Fixation and Permeabilization buffers (Invitrogen, Waltham, MA) and stained with intracellular antibodies for 60 minutes at 4°C. Antibodies used were anti-human Ki-67 PE-Cy7 (clone B56, 561283; BD Biosciences), EOMES APC-eF780 (clone WD1928, 47-4877-42; eBioscience), KIR3DL1 BV421 (clone DX9, 312714; BioLegend), Granzyme K PerCP-Cy5.5 (clone GM26E7, 370514; BioLegend), and KIR2DL4 AF700 (clone 181703, FAB2238N-100UG; R&D Systems, Minneapolis, MN). Measurement was performed on a FACSCanto (BD Biosciences) or LSR Fortessa (BD Biosciences) (for gating strategy see Figure 7).

For sorting, the intestinal cells were incubated with the surface antibodies for 20 minutes in supplemented RPMI (2% FCS, 1% penicillin and streptomycin, 0.2% Fungizone) at 4°C, and subsequently washed in fluorescence-activated cell sorting buffer before sorting on a FACSAri III (BD Biosciences) (for gating strategy see Figure 7). Antibodies used were fixable viability dye eF506 (65-2860-40; eBioscience), anti-human TCRγδ BV510 (clone B1, 331220; BioLegend), CD3 AF700 (clone UCHT1, 300424; BioLegend), CD4 BV785 (clone OKT4, 317442; BioLegend), CD8α APC-Cy7 (clone SK1, 557834; Beckman Coulter, Brea, CA), CD127 BV421 (clone HIL-7R-M21, 562436; Beckman Coulter), CD25 PE-Cy7 (clone M-A251, 557741; Beckman Coulter), CD69 PE (clone FN50, 555531; BD Biosciences), CD103 FITC (clone 2G5, 550259; Beckman Coulter). Flow data were analyzed using FlowJo v10 (TreeStar, Ashland, OR).

### RNA-Sequencing

The sorted cells were thawed for TRIzol (Thermo Fisher Scientific) RNA extraction and stored at –80°C until library preparation. Sequencing libraries were prepared using the Cel-Seq2 Sample Preparation Protocol and sequenced as 75 bp paired-end on a NextSeq 500 (Utrecht Sequencing Facility). The reads were demultiplexed and aligned to the human complementary DNA reference genome (hg38) using BWA (version 0.7.13; http://bio-bwa.sourceforge.net/). Multiple reads mapping to the same gene with the same unique molecular identifier (6 bp long) were counted as a single read.

Raw counts of splice variants were summed and the raw counts were subsequently transformed employing variance stabilizing transformation. Ensembl names were converted to HGNC symbol, and if no symbol had been assigned the ensembl reference name was used. Differential analysis was performed using DESeq2 (Wald’s test). For visualization purposes, the R version 4.0.0 packages DESeq2, EnhancedVolcano, and pheatmap were employed (R Foundation for Statistical Computing, Vienna, Austria). Raw counts were used as input for generating volcano plots with the genes colored based on *P* value and log2 fold-change cutoffs, with selected gene symbols shown for the genes with an FDR < 0.1. For heatmaps, transformed counts were *z* score normalized followed by hierarchical clustering based on samples and genes. Pathway analysis was performed on the differentially expressed genes as input in ToppFun with standard settings. Gene set enrichment analysis, with as input the normalized data (output DESeq2), was used to assess enrichment of gene sets derived from the MSigDB C7 database (immunological signatures), and the Trm cell signature for human CD8^+^CD69^+^CD103^+^ T cells as defined by Hombrink et al^25^. One thousand random permutations of the phenotypic subgroups were used to establish a null distribution of enrichment score, against which a normalized enrichment score and FDR-corrected *q* values were calculated. RNA-sequencing data are available at GEO Accession GSE160925.

### Statistical Analyses

Flow cytometric data were analyzed with the independent 1-tailed or 2-tailed (paired) *t* test, or with a 1-way analysis of variance with post hoc Tukey’s. For correlation analysis, Spearman’s correlation was used. Data were analyzed with SPSS Statistics version 22.0.0.0 (IBM, Armonk, NY) and GraphPad Prism version 7.0 (GraphPad Software, San Diego, CA).

## Ethics Approval

The study protocols (NL28761.091.09 and TCBio 17/443, 17/444, 18/522) were approved by the research ethics committee of the Radboud University Nijmegen Medical Centre (CMO Regio Arnhem-Nijmegen, Nijmegen, the Netherlands) and the University Medical Center Utrecht, respectively. Written informed consent was obtained from each participating patient before any study-related procedure was performed. The procedures were performed in accordance with the Declaration of Helsinki.

All authors had access to the study data and reviewed and approved the final manuscript.
